# Hyperconnectivity during screen-based stories listening is associated with lower narrative comprehension in preschool children exposed to screens vs dialogic reading: An EEG study

**DOI:** 10.1371/journal.pone.0225445

**Published:** 2019-11-22

**Authors:** Rola Farah, Raya Meri, Darren S. Kadis, John Hutton, Thomas DeWitt, Tzipi Horowitz-Kraus

**Affiliations:** 1 Educational Neuroimaging Center, Faculty of Education in Science and Technology, Technion, Haifa, Israel; 2 Division of Neurology, Cincinnati Children’s Hospital Medical Center, Cincinnati, Ohio, United States of America; 3 Pediatric Neuroimaging Research Consortium, Cincinnati Children’s Hospital Medical Center, Cincinnati, Ohio, United States of America; 4 Department of Pediatrics, College of Medicine, University of Cincinnati, Cincinnati, Ohio, United States of America; 5 Reading and Literacy Discovery Center, Cincinnati Children’s Hospital Medical Center, Cincinnati, Ohio, United States of America; University of Trento, ITALY

## Abstract

**Objectives:**

Dialogic reading (DR) is a shared storybook reading intervention previously shown to have a positive effect on both literacy and general language skills. The aim of this study was to examine the effect of DR compared to screen-based intervention on electrophysiological markers supporting narrative comprehension using EEG.

**Methods:**

Thirty-two typically developing preschoolers, ages 4 to 6 years, were assigned to one of two intervention groups: Dialogic Reading Group (DRG, n = 16) or Screen Story Group (SSG, n = 16). We examined the effect of intervention type using behavioral assessment and a narrative comprehension task with EEG.

**Results:**

The DRG showed improved vocabulary and decreased functional connectivity during the stories-listening task, whereas the SSG group showed no changes in vocabulary or connectivity. Significantly decreased network strength and transitivity and increased network efficiency were observed in the DRG following intervention. Greater network strength and transitivity at follow-up were correlated with increased vocabulary.

**Conclusions:**

The results suggest the beneficial effect of DR in preschool-age children on vocabulary and EEG-bands related to attention in the ventral stream during narrative comprehension. Decreased functional connectivity may serve as a marker for language gains following reading intervention.

**Significance:**

DR intervention for preschool-age children may reduce interfering connections related to attention, which is related to better narrative comprehension.

## Introduction

### How does a child learn to read?

Reading is a fundamental skill for knowledge acquisition that is vital for academic and vocational success, social function, and health [1–3]. As reading is evolutionarily new (approximately 5,400 years), there are no innately-specified networks specific to reading [4, 5]. Beginning in infancy, brain areas and networks adapted for other functions such as vision (visual word form area, ventral stream) [6] and language (phonetic processing: bilateral superior temporal gyrus; meaning of sounds: left posterior middle temporal gyrus; production: posterior inferior temporal gyrus, aka the ventral stream [7]; mapping visual characters into sounds or decoding: angular gyrus and supramarginal gyrus (dorsal stream) [8]) and higher-order abilities such as working memory (superior temporal gyrus) [7] and executive functions [9] are gradually synchronized along development even prior to reading age when the dorsal and ventral streams become functionally connected [10]. These neural circuits then become integrated in response to reading exposure and practice [5]. This neurobiological process underlies emergent literacy, which is based on the skills, knowledge, and attitudes required to learn to read and write [11], and its foundation is laid during the span of rapid brain growth between birth and age 5 years [12] (for review see [13]). The older children become and the more they are exposed to written language, they shift their reliance toward the left hemisphere [6, 14], and more specifically from the left dorsal stream to the ventral stream, while reading [8]. The increased utilization of the ventral stream along development has been related to several changes that occur with increased age: reading exposure related to increased activation in the left middle temporal and inferior frontal gyri and increased phonological abilities related to activation in the left posterior superior temporal sulcus in children [15].

Exposure to literacy material at a young age (preschool) is one way to trigger the involvement of the ventral stream, and more specifically the occipital cortex [16]. Hutton and colleagues showed that greater home literacy exposure, including number of books in the house and joint reading time, is positively related to the activation of the occipito-temporo-parietal junction while listening to stories [16]. This activation was related to imagination and visualization during listening to stories, which are facilitated by exposure to literacy at a young age. One approach to facilitate exposure to written material is shared reading [16]. As early as at the age of 6 months, shared reading has been related to improved cognitive, language, and social emotional abilities at 12 months of age [17]. Frequent shared storybook reading is known to lead to vocabulary growth and in turn, later success in reading and other academic areas [18–22]. It is therefore not surprising that maternal shared reading quality was associated with greater activation in neural circuits related to executive functions (frontal pole), language processing (inferior frontal gyrus), and socio-emotional abilities (anterior insula) during stories listening in 4-year-old girls [23]. Others have shown that maternal reading fluency is related to greater functional connections while listening to stories between neural circuits that will form the reading network during reading age [24]. These neural circuits included the ventral stream related to word recognition and phonological processing [24], which emphasizes the importance of the quality of reading a child is exposed to during preschool ages. A well-validated shared storybook reading intervention is called Dialogic Reading (DR). One question addressed in the current study was whether a DR intervention would be associated with greater involvement of occipital regions in preschool-age children.

### Dialogic reading intervention

DR uses a set of standardized prompts to explicitly target a young child's oral vocabulary and listening comprehension skills [25, 26]. The process involves asking open-ended questions about the story, expanding on the child's answer by repeating it, clarifying the question or asking further questions, and offering praise and encouragement to the child for giving input into the story [11].

Reading dialogically to children at risk for academic failure increases both their expressive and receptive language [11]. However, the specific mechanism for this change remains unknown. Since one of the key components in DR is the direct involvement of the child during storytelling, a recent study examined the effect of DR on executive functions in preschool children [27]. The researchers demonstrated that children in the group exposed to DR (DRG, in the current study) showed higher executive functions abilities compared to those in the control group who was exposed to the same story presented on the screen (SSG, in the current study). The researchers suggested that direct attention and stimulating questions during the DR process triggered joint attention, which was previously reported to be crucial for learning [28–31]. However, the involvement of occipital regions critical for future reading acquisition during stories listening following DR was yet to be determined.

### Narrative comprehension, what and how

The ability to listen and comprehend orally presented language is termed narrative comprehension, an ability that emerges early in life [32, 33]. The importance of this ability is demonstrated by the critical role of stories listening in language development [34, 35] and, later in life, in reading acquisition and comprehension [36]. The ability to comprehend a narrative relies not only on speech perception, auditory word recognition, syntactic processing, and discourse coherence [37], but also on neural circuits related to higher-order cognitive abilities - working memory and executive functions [36]. This is supported by previous neuroimaging studies examining stories listening in children. A functional magnetic resonance imaging (fMRI) stories-listening task (i.e., listening to a series of multiple stories) in 5-18 year-old participants engaged neural circuits known to support language skills – inferior frontal gyrus [Brodmann Area (BA) [44,45], superior temporal gyrus (BA 22), and angular gyrus (BA 40,39) – and also frontal regions (BA 10,6,46,9,8) that likely were related to the involvement of executive functions in this task [36]. The involvement of neural circuits supporting narrative comprehension in a fine temporal resolution can be examined using event-related designs (see [38, 39]), as well as in the phase level. The latter enables an examination of the involvement of neural circuits during narrative comprehension for a longer period of time (i.e., not only for specific “events”) that depends on the time window determined. Using magnetoencephalography (MEG) recording during sentences listening in six English-speaking adults, an increased theta power (4-8 Hz) was demonstrated in the auditory cortex [40]. Participation of high frequency power (> 30 Hz) in both language and attention tasks has been shown in adults [41].

Interestingly, the electrophysiological markers for language and attention processes change along development with several studies suggesting either an increase or decrease in power along development during rest or task conditions. Some report of a better language and attentional performance related to high theta and lower alpha in younger children and low theta and increased alpha in adults during task [42]. During rest, however, a typical increase in alpha is observed during 3 to 9 years of age (see [43–45]), as also seen in adults [46, 47]. This aligns with the findings that higher interhemispheric alpha during rest is related to increased academic abilities in adults [46]. The increased alpha along age was related to the cognitive resources allocated to inhibit responses for irrelevant stimuli (better focus on the task) [44, 48], suggesting an alternate ‘neural strategy’ as the task becomes effortless through mastery. Alpha power was also related to processing of sensory stimulation; i.e., increased alpha for an auditory stimulation (not a verbal one) that was not observed in visual processing regions, and vice versa [45]. Altogether, these results indicate the feasibility of detection of spectral power in young individuals during rest as well as during a task and how it is specific to the ability to allocate attention to a specific stimulus (and modality) and inhibit responses to others. Therefore, we suggest that relative to a SSG, the DRG who will be exposed to stories delivered by an experimenter will show a reduced level of need to inhibit task-irrelevant stimuli and therefore, will show decreased alpha oscillations that are inversely correlated with cognitive performance [48] in posterior regions. This may represent a decreased effort focusing on the story and inhibit irrelevant stimuli.

The current study aimed to reveal the effect of DR intervention on neural circuits supporting the ventral stream using EEG; i.e., visual processing during narrative comprehension related to visualization of the presented auditorily linguistic stimuli. We predicted that DRG would show greater narrative comprehension scores in the EEG task and general language skills, compared to the SSG. We also predicted that the DRG would demonstrate less functional connectivity in alpha frequency range, related to the ability to inhibit task-irrelevant cortical structures [48], in occipital regions (i.e., ventral stream) related to visual attention in EEG data acquisition during stories processing.

## Materials and methods

### Participants

The study participants were thirty-two native-Hebrew speaking 4-6 year-old children (13 females) from a middle-class background; all were right-handed, had normal or corrected-to-normal vision in both eyes, were found to have normal hearing, and had a normal neurological and developmental history. This information was initially verified over the phone while communicating with the parents upon enrollment into the study.

Participants were randomly assigned to two intervention groups: DRG (n = 16) or SSG (n = 16). The two groups did not significantly differ in age (DRG: mean age = 64.35 months, standard deviation = 3.99 and SSG: mean age = 58 months, standard deviation = 6, t(31) = 2.246, n.s.) or their attention/hyperactivity scores [*t*(31) = 1.749, n.s.] as measured by the Conners Rating scale [49]. See Table 1.

**Table 1:** Differences between the dialogic reading and screen stories intervention groups before intervention using independent-samples *t* tests.

Measures	DRG	SSG	T	Significance
	Mean (SD)	Mean (SD)		
Age	63.35 (3.99)	58 (6)	2.246	*P* = 0.23
Attention abilities (Conners, T score),	87.49(5.72)	83.05(9.78)	1.749	*P =* 0.1
Nonverbal ability (Matrix, WPPSI, scaled score), average range: 7-13	10.44 (2.61)	10.31 (3.12)	0.135	*P* = 0.893
Verbal ability (Naming, WPPSI, scaled score), average range: 7-13	9.11 (2.51)	9.11 (2.74)	0.000	*P* = 1
Vocabulary (WPPSI, scaled score), average range: 7-13	10.33 (2.44)	9.78 (2.87)	0.617	*P* = 0.514

DRG, dialogic reading group; SSG, screen story group; SD, standard deviation; WPPSI, Wechsler Preschool and Primary Scale of Intelligence

Parents of the participants provided written informed consent prior to the study. All participants attended a preschool in the north of Israel. The study was reviewed and approved by the Technion’s Institutional Review Board (approval: 082016).

### Procedures

Participants were assessed within their day care facility. Tests were randomized for order of delivery and administered individually in one of the schoolrooms during school hours by the research team prior to and after intervention. EEG data was acquired after the intervention in a sound-attenuated room in the Educational Neuroimaging Center at the Technion, Israel. As the reception room is decorated with child-friendly toys, the study team made sure children were comfortable entering the EEG testing room. Parents accompanied the children to the testing session. Each child had the opportunity to explore the surroundings and play while the EEG cap was connected and a gel was placed in the electrodes. The experimenter explained to the child that a story would be played and he/she would be asked to listen to the story and then some questions would be asked (to verify comprehension). Then, the child heard a story as an example. Only after following these steps was the EEG data acquisition started. The study team was trained and practiced the DR sessions following [26], read further for details. Questions for each story were prepared and practiced before the sessions in the daycare. For the screen-story condition, the books were orally read by the same experimenter administering the DR intervention. While the book was being read, only the pages of the book were videotaped (the experimenter was not videotaped).

### Interventions

Both groups participated in eighteen, 30-min long sessions of dialogic storytelling sessions over the course of 6 weeks, 3 times per week. The research team was trained on DR using several steps for structured DR intervention [26].Each DR session included the following: 1) Prompt the child to say something about the story, 2) Evaluate what the child says, 3) Expand on what the child says, and 4) Repeat and reinforce associations; PEER. Similarly, the acronym CROWD reflects evocative parental or caregiver prompts: 1) Completion of a sentence, 2) Recall earlier aspects of the story, 3) Open-ended questions, 4) *W**h-* questions, and 5) Distancing by relating the story to the child’s experience. Books were age-appropriate, different in each session, and provided by the PJ-library organization. To better allow the children’s participation, each intervention group (i.e., DRG, SSG) was divided into two smaller groups of eight children each.

The SSG was presented with the exact same stories to which the DRG was exposed. The stories from the books were recorded while being read by the same experimenter who read the stories to the DRG. Then, the SSG watched these videos, without any interaction with the experimenter or any questions asked during or after the stories were read.

### Behavioral and experimental measures

To assess linguistic ability in the DRG and SSG following intervention, vocabulary (i.e., picture naming [50]) and objects/colors rapid naming abilities [51] were assessed before and after intervention in the two groups. Tests for general verbal and nonverbal abilities [50] were administered. Each behavioral testing session lasted approximately 1.5 hours.

#### Electrophysiology recordings

Stories-listening task. To test the effect of intervention on neural circuits supporting narrative comprehension abilities, a stories-listening task (i.e., listening to a series of multiple stories) was administered following [52]. Stories were recorded in a sound-attenuated room using a professional recording microphone. Through speakers, the participants listened to Hebrew stories taken from age-appropriate books. Five 30-second stories were alternated with five backwards-presented speech conditions, 30 seconds each. While listening to each story, a cross was presented on the screen. Participants were instructed to listen to the stories and answer five multiple-choice comprehension questions at the end of the task. The questions were presented auditorily by the experimenter, and the participant responded orally.

EEG recording. The participant was seated approximately 80 cm in front of an IBM-PC screen in a sound-attenuated room and presented with the stories through speakers. The overall task lasted 5 minutes and was played using an eprime system (Psychology Software tools (Sharpsburg, PA, USA). EEG data was recorded continuously using the PyCorder software from 64 actiCHmpt electrodes mounted on a custom-made cap (Brain Products, Gmbh; EasyCap, Germany) according to the international 10/20 system, sampled at a rate of 500 Hz with an analogue band pass filter of 0.1 Hz to 70 Hz and 12-bit A/D converter using a Dell PC (Round Rock, Texas, USA), and stored for off-line analysis. A ground electrode was placed at the front of the cap (below the AFz electrode) and an average reference method was applied. All electrode impedances were maintained at or below 5 KΩ.

#### Data analyses

EEG data preprocessing. EEG analyses were carried out using primarily FieldTrip [53] routines running in MATLAB (version R2016a; WathWorks Inc., Natick, MA). We adopted a minimal-scrubbing approach since the impact of artifact suppression (filtering) on source-space connectivity analyses is unknown, and since beamformer inversions for induced effects are generally robust to brief artifactual signals. Raw data were average referenced across sensors. Continuous recordings were initially bandpass filtered from 0.5 Hz to 70 Hz, and power line noise was attenuated at 50 Hz using a very sharp, discrete Fourier transform filter. The initial 26 seconds of each story trial were extracted from the continuous recording, appended, then segmented into 68 discrete 2-second epochs for subsequent analyses.

Source analysis, virtual sensors. A generic boundary element head model (BEM) [54] was used for all subjects. The BEM was constructed from the MNI-152 template, representing a non-linear average of 152 T1-weighted scans of healthy adults [55].To promote comparison in our developmental cohorts, a single, robust adult-derived template was preferred to head models built from multiple age-specific MRI surrogates. Electrode positions were automatically aligned and projected to the outer surface of the scalp compartment of the BEM. We constructed a regular 3D grid with 20 mm dipole spacing inside the cerebral compartment and estimated the time series of activity at each grey-matter location using a linearly constrained minimum variance (LCMV) beamformer with 5% regularization [56]. The LCMV beamformer is a spatial filtering technique, whereby signal at a specified location is passed with unit gain while signals originating from other locations are suppressed (null gain). Sources were estimated in canonical directions at each position in our grid and projected along the strongest orientation. The resulting ‘virtual sensor’ time-courses were then analyzed for pairwise functional connectivity.

Connectivity analyses. We computed spectrally-resolved (2 Hz to 70 Hz, in 0.5 Hz bins) phase-based functional connectivity between all virtual sensor pairs (network nodes) using a debiased weighted phase-lag index (wPLI) (see [57]). The debiased wPLI metric is computed from the cross-spectrum for a pair of signals, obtained from conjugate multiplication of their Fourier representation. The absolute value of debiased wPLI was summed for all nodes pairs, per subject, at each frequency bin. We then inspected the connectivity spectra for each group and ran independent-samples *t*-tests at each frequency to identify possible contiguous bands of functional connectivity differences.

Network-based analyses. For frequencies showing reliable group differences in connectivity, we computed mean absolute debiased wPLI at each node, per participant, and compared group network topologies using Network Based Statistical (NBS) analyses [58]. The NBS approach is entirely data-driven and circumvents multiple comparison problems by comparing observed differences in network extent (number of connected nodes, at an arbitrary initial threshold) to those obtained from permuted distributions. We tested the observed network cluster extent against 5,000 permutated distributions, using an alpha of 0.05 at a range of (arbitrary) initial thresholds greater than 1. Significant group differences were visualized at a median, representative initial threshold.

We also describe whole-brain connectivity differences using two graph-theoretical summary metrics. Network *transitivity* (i.e., clustering [59]) and *global efficiency* [60], which are common measures of segregation and integration, respectively (see [61] [62]), were calculated for each group across all sensors in source space and compared using independent-samples *t*-tests. Where group differences were observed, we assessed the correlation between network measures and behavioural performance.

Statistical analyses. To rule out between-group differences at baseline, behavioral data prior to intervention were analyzed using independent-samples *t*-tests analyses. To test the effect of intervention between and within each group, 2x2 [Group (DRG, SSG) x Test (before, after intervention)] Repeated measures ANOVAs were conducted for each behavioral measure and corrected for multiple comparisons, followed by post hoc paired *t*-test analyses. Differences in EEG connectivity as well as ability to comprehend the stories between the two groups were determined using independent-samples *t*-tests analyses.

Correlations between the behavioral gains (i.e., the difference between Test 2 and Test 1 for vocabulary and naming abilities) and between the behavioral accuracy percentage following the narrative comprehension task (ability to understand the stories), as well as the connectivity for each group were computed. The data were corrected using Family-wise Type-I error rates by the Bonferroni method for multiple comparisons (ά<0.05).

## Results

### Behavioral measures

#### Behavioral baseline measures

In general, all participants showed average and above average general nonverbal and verbal abilities. At baseline (Test 1), participants in the two groups did not differ in linguistic and literacy abilities. See Table 1.

#### Behavioral measures, the effect of intervention

Repeated measures ANOVAs demonstrated a main effect of Test for the verbal vocabulary measure (F(1,31) = 7.547, *P* = 0.009, ή^2^ = 0.177); increased vocabulary abilities were observed following intervention. No significant main effect was found for Group for the interaction [Group, F(1,31) = 0.193, *P* = 0.663, ή^2^ = 0.005, Test x Group F(1,31) = 0.827, *P* = 0.369, ή^2^ = 0.023]. No significant Group effect or interaction was observed for the linguistic tests [Test: F(1,35) = 2.159, *P* = 0.151, ή^2^ = 0.058; Group: F(1,31) = 0.088, *P* = 0.769, ή^2^ = 0.003; Test x Group: F(1,31) = 0.358, *P* = 0.553, ή^2^ = 0.010]. Follow-up tests of simple effects (paired *t*-test analyses) demonstrated a significant improvement in vocabulary score but not in naming following intervention in the DRG compared to the SSG. See Table 2 for these results as well as S1 File.

**Table 2:** Effect of intervention in dialogic reading and screen story intervention groups on linguistic measures.

Behavioral/Cognitive testing	DRG		SSG			T (*P*)
	Test 1	Test 2	Test 1	Test 2	Condition	
	Mean (SD)	Mean (SD)	Mean (SD)	Mean (SD)		
Verbal ability (Vocabulary, WPPSI, scaled score) average range: 7-13	9.11 (2.51)	10.315 (2.08)	9.11 (2.74)	9.42 (3.59)	DRG, Test 2>Test 1	-2.483 *P* = 0.023
					SSG, Test 2>Test 1	-1.368 *P* = 0.189
					DRG, Test 1 = SSG, Test 1	0.000 *P* = 1
					DRG, Test 2>SSG Test 2	0.939 *P* = 0.354
Objects/colors rapid naming (Shatil, scaled score) average range (-1)-(+1)	-0.166 (2.065)	0.333 (1.748)	-0.21 (2.299)	0.0 (2.108)	DRG, Test 2>Test 1	-1.767 *P* = 0.095
					SSG, Test 2>Test 1	-0.544 *P* = 0.593
					DRG, Test 1>SSG Test 1	-0.147 *P* = 0.884
					DRG, Tes2 1>SSG Test 2	0.522 *P* = 0.605

DRG, dialogic reading group; SSG, screen stories group; SD, standard deviation; WPPSI, Wechsler Preschool and Primary Scale of Intelligence

#### Electrophysiological data

Stories listening; behavioral measures. No differences were found between the groups for narrative comprehension following intervention (see Table 3).

**Table 3:** Differences in behavioral and network (EEG) measures for the stories-listening task between the dialogic reading and screen stories intervention groups.

Measures	DRG	SSG	T	*P* value
	Mean (SD)	Mean (SD)		
Behavioral measures				
Accuracy rate - stories listening (percent)	68.75 (30.95)	65.88 (27.17)	-0.283	0.78
EEG measures				
Network strength	15.01 (3.95)	80.83 (57.06)	-4.41	0.001
Network efficiency	0.86 (0.38)	0.13 (0.07)	-2.227	0.03
Network transitivity	0.03 (0.01)	0.05 (0.03)	-2.234	0.03

DRG, dialogic reading group; SSG, screen story group; SD, standard deviation

**Fig 1:**
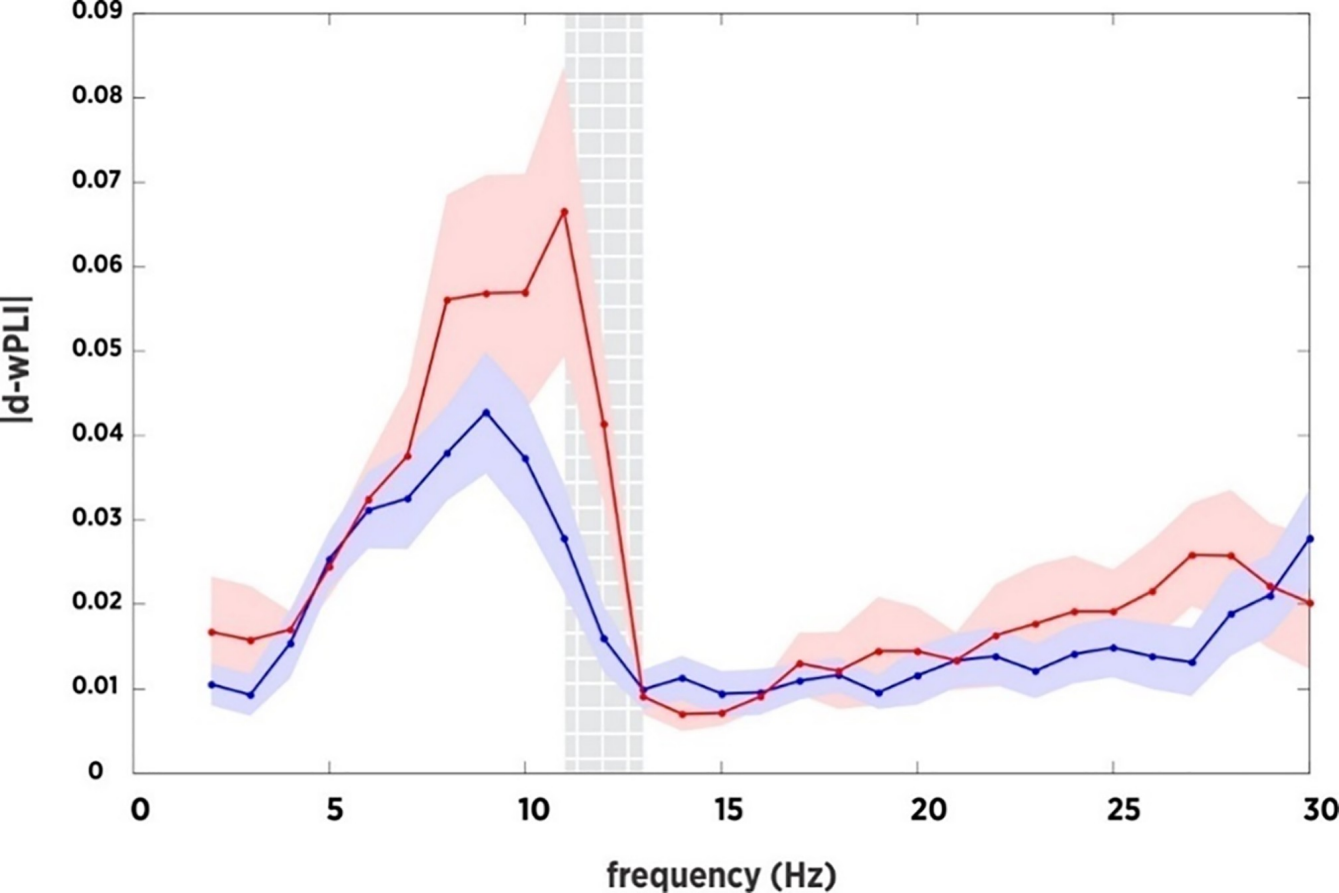
Differences in alpha functional connectivity between the dialogic reading and screen stories intervention groups. The screen stories group (red) showed significantly increased alpha functional connectivity (debiased wPLI) compared to the dialogic reading group (blue). A contiguous band of increased connectivity was observed between 10 and 13 Hz, in 0.5 Hz increments.

**Fig 2:**
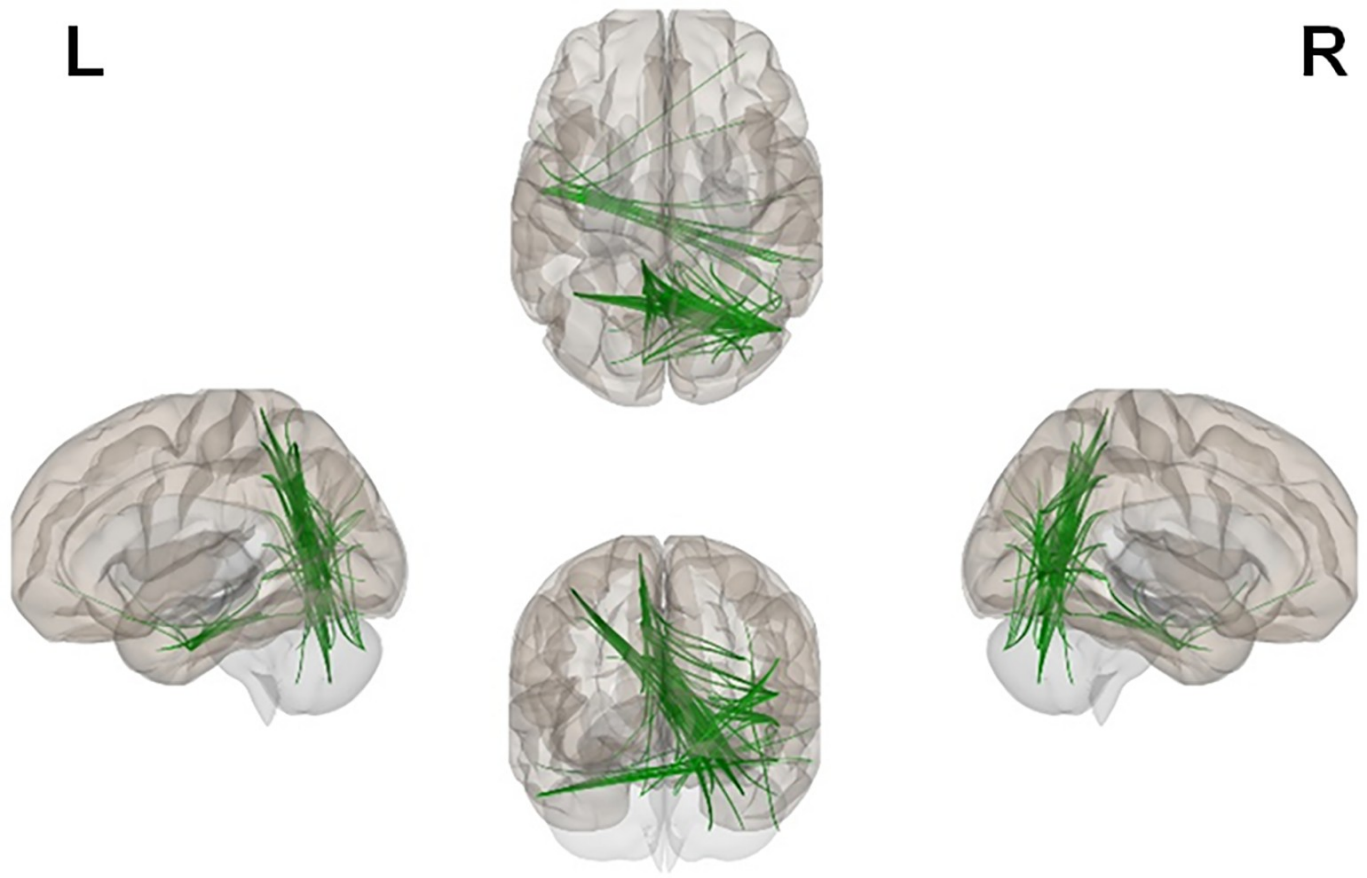
A comparison in functional connectivity between the dialogic reading and screen stories groups (screen>dialogic reading). The screen stories group showed a single cluster of increased posterior functional connectivity during stories listening. Findings from NBS analyses; 5,000 permutations, *P*<0.05, at initial thresholds of t = 2 through 4 at increments of 0.2. Shown here, the cluster of increased connectivity at t = 3.2. (Neurological orientation, L = left, R = right).

Connectivity spectra and NBS findings. We observed significant group differences in the connectivity spectra. Specifically, the SSG showed significantly increased whole-brain functional connectivity at 10-13 Hz (approximately alpha band). NBS for 10-13 Hz connectivity revealed a single cluster of increased functional connectivity in the SSG (5,000 permutations, alpha = 0.05, *t* = 2 to four initial thresholds) compared to the DRG. The cluster of connections was plotted in brain space and revealed posterior interhemispheric hyperconnectivity in the SSG compared to the DRG. See Table 3 and Figs 1 and 2 for these results.

#### Whole-brain graph analyses

Significant lesser network strength was observed in the DRG compared to the SSG [t(30) = -4.41, *P* = 0.001].

Significant greater network efficiency [t(30) = -2.227, *P* = 0.03] and lesser network transitivity [t(30) = -2.234, *P* = 0.03] were observed in the DRG compared to the SSG. Results are reported in Table 3.

**Fig 3:**
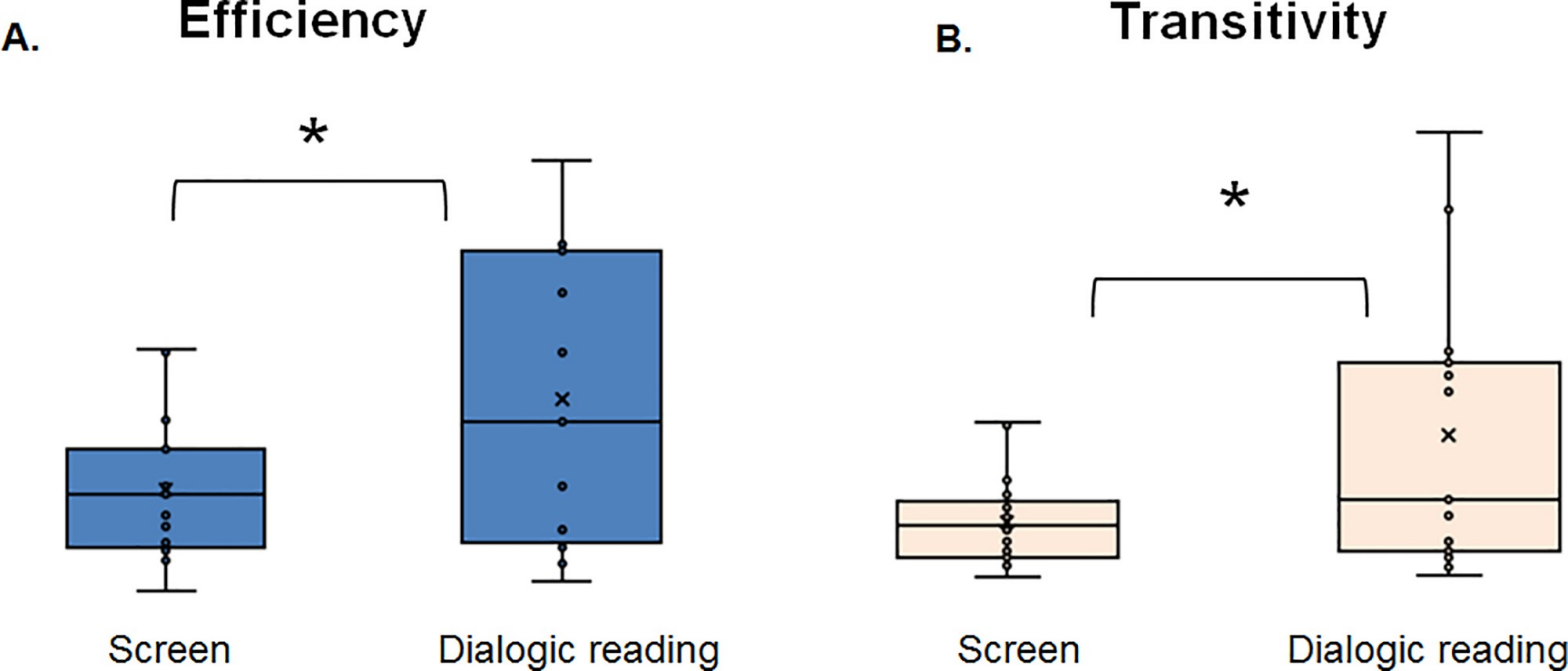
Network’s transitivity and efficiency in the dialogic reading and screen stories intervention groups. (A) Increased network’s transitivity (left panel) and (B) efficiency (right panel) for the screen stories group (Screen; right bar in each panel) compared to the dialogic reading group (Dialogic reading; left bar in each panel). *P<0.05.

Pearson correlations between the EEG network measures and the stories-listening accuracy measures across the two intervention groups revealed significant correlations between accuracy and network strength (r = -0.4, *P*<0.05) and between accuracy and network transitivity (r = -0.34, *P*<0.05). Greater network strength and transitivity were related to a lesser ability to understand the stories. No significant correlations between accuracy rate for stories listening and efficiency measures were found in the two intervention groups (r = -0.29, *P* = 0.122). See Fig 3.

## Discussion

The aim of the current study was to examine the effect of DR intervention compared to screen-based stories listening on neural networks related to comprehending stories during a narrative-comprehension EEG-based task. In support of our hypotheses, the DRG showed improved vocabulary abilities that was absent in the SSG. Also, lower network strength and transitivity during stories listening were related to higher comprehension of the stories presented during the EEG session, both of which were observed in the DRG.

The ventral stream is triggered in preschool children during dialogic reading

The results of the current study reveal the involvement of the ventral stream during DR. As children learn how to read, the ventral stream is involved in the reading process [6]. Several studies have indicated the importance of narrative comprehension and stories listening at pre-reading age, with evidence of the involvement of parts of the ventral stream (i.e., occipital activation) while listening to stories [16, 36]. The assumption was that children imagining the stories activates these occipital regions. In the current study, we found that the combination of both telling stories and actively engaging the child in the storytelling process in a dialogic way was associated with lower network strength in the DRG, which was overall related to better narrative comprehension. We postulate that the joint attention while telling stories in the DRG demonstrates a reduced need to avoid distraction during storytelling (following [3, 44]) delivered by the experimenter. The better comprehension associated with this lower network strength and transitivity during stories listening may contribute to the preparation for the involvement of the ventral stream during this pre-reading period.

### Dialogic reading method supports the “outside-in” component in the inside-out, outside-in model

The results of the current study suggest that it is not only the exposure to a verbal stimulation that is critical for a child’s language development, but also the interaction around the book as suggested in the outside-in and inside-out mode [11]. As proposed in this traditional model, the interaction around the book involving questions, brain storming, and pulling out information from long-term memory and general knowledge (prompted by the adult’s questions) is important for the facilitation of future reading ability. We found that the DRG showed less alpha connectivity involved during stories listening compared to the SSG. This difference in alpha connectivity was related to better attention abilities in the DRG while listening to stories compared to the SSG. As suggested by the outside-in model, the exposure to narratives in a dialogic interactive way leads to a more automatic verbal processing, allowing the child to devote attention to other aspects of the story (e.g., conclusions, inference). However, it will be interesting to further investigate whether this change in alpha in the DRG extends to non-verbal conditions as well or is specific to when listening to stories. A further study looking at a resting-state condition after DRG vs SSG should examine this point in depth.

Human interaction around the book results in more efficient language processing

A possible explanation for the positive effect of DR on vocabulary may be related to Kuhl’s studies [12, 30, 31, 63] that suggest directed speech and language. Although these studies focus on speech and language acquisition, and not on early literacy skills, the researcher also emphasized the critical role human interaction has on acquiring these abilities. Kuhl postulates that direct interaction with an adult increases the child’s attention and improves the ability to capture the new linguistic stimuli provided. This effect was not seen when children were exposed to the same stimuli provided on a television screen [30, 31]. Not only does the child need exposure, but a crucial step in increasing the child’s linguistic abilities is eye contact, and change in tone is needed, all of which is summarized as “social gate” as termed by Kuhl [12, 29–31, 63, 64]. In her Native Language Magnet theory-expanded model (NLM-E), Kuhl explains how language exposure and social interaction merge into what we see as language acquisition, through joint attention both to the auditory aspect (‘motherese’) and the visual aspect (object-sound relationships) [65]. These findings support EEG results showing greater power (focusing on theta band) during an infant-directed speech compared to adult-directed speech in 7 month-old children [66]. These findings suggest that a directed communication with infants using speech-tones and words that young infants are sensitive to, similar to motherese, has neural correlates for this sensitivity.

A previous study looking at the effect of DR on visual attention and language ability confirmed this as well, by demonstrating elicited P300 component during the Attentional Network Task (ANT) in a DRG [27]. As a task utilizing the attentional network, the ANT is capable of demonstrating an improvement in both orienting attention (measured by the P300) in the DRG. Our study is the first to show a direct effect of DR intervention on neural networks related to language processing and specifically to learning new works (i.e., vocabulary) during stories listening. Similar to previous other studies [42, 67] the DRG of the current study showed improved vocabulary and changes in brain patterns related to the ability to focus on a relevant stimulus or avoid destruction during stories listening relative to the SSG, as demonstrated by the decreased alpha connectivity in this group that previously has been related to better attention abilities to a given task [68, 69].

### Study limitations

As a pioneering study designed to explore the neurobiological mechanisms for the involvement of the ventral stream in pre-reading age after DR, the EEG data were collected only after the intervention, which poses a limitation on the results of the current study. Although we have demonstrated a connection between comprehension of stories following the EEG task and network strength, a future study including EEG data collection prior to the intervention should be conducted. To specifically pinpoint neural circuits related to language processing and relate the presumably changes in attention to those in narrative comprehension, a functional MRI study looking specifically at the functional connectivity between the language and cognitive-control network should be conducted. Additionally, the current study’s results suggest significant changes following intervention (found in the t-test analysis), but no significant interaction in the ANOVA. This discrepancy might be the consequence of our relatively smaller sample. An additional study including a larger sample should be conducted to determine the significant effect in one intervention vs the other. Moreover, to rule out the effects of typical development on our results, a control group of children who do not participate in the intervention, but who are tested at both Test 1 and Test 2 should be included. Another important point to specify the effect of DR on neural circuits supporting language development, is the need for an additional control group of an adult telling stories to children without a dialogue. This control group is extremely challenging to employ, as children naturally ask questions while interacting with an adult around stories, however is important to consider. Lastly, to account for the effects of social interaction and the in-depth questions involved in the DRG, an additional group of children exposed to the DR questions on the screen should be added.

## Conclusions

The current study was the first to provide neurobiological support for the effect of DR intervention on neural circuits supporting language processing, pointing at the involvement of the ventral path in language processing following DR. Due to the specific frequencies tested in this study (alpha band) during stories listening, the results provide an initial step towards a mechanistic understanding of the effect of a dialogue that also involves brain synchronization related to attention. Future studies are needed to determine the generalization of these results to domains other than language (i.e., during rest) in older individuals and also younger children when language and attention abilities.

## Supporting information

S1 File Behavioral and connectivity data. (XLS)

## References

[1] High PCK. Literacy promotion: an essential component of primary care pediatric practice. Pediatrics. 2014;134:404–9. 10.1542/peds.2014-1384 24962987

[2] Dewalt DA, Berkman ND, Sheridan S, Lohr KN, Pignone MP. Literacy and health outcomes: a systematic review of the literature. Journal of General Internal Medicine,. 2004;19(12):1228–39. 10.1111/j.1525-1497.2004.40153.x 15610334 PMC1492599

[3] Green CM, Berkule SB, Dreyer BP, Fierman AH, Huberman HS, Klass PE, et al Maternal literacy and associations between education and the cognitive home environment in low-income families. Archives of Pediatric and Adolescent Medicine. 2009;163(9):832–7. 10.1001/archpediatrics.2009.136 PMC3083977 19736337

[4] Dehaene S. Reading in the brain: The new science of how we read. New York, NY: Penguin; 2009.

[5] Dehaene S, Cohen L. Cultural recycling of cortical maps. Neuron. 2007;56(2):384–98. Epub 2007/10/30. 10.1016/j.neuron.2007.10.004 . 17964253

[6] Schlaggar BL, McCandliss BD. Development of neural systems for reading. Annual review of neuroscience. 2007;30:475–503. 10.1146/annurev.neuro.28.061604.135645 . 17600524

[7] Ben Shalom D, Poeppel D. Functional Anatomic Models of Language: Assembling the Pieces. tHE Neuroscientist. 2008;14:119–27. 10.1177/1073858407305726 17911215

[8] Sandak R, Mencl WE, Frost J, Pugh KR. The Neurobiological Basis of Skilled and Impaired Reading: Recent Findings and New Directions. SCIENTIFIC STUDIES OF READING. 2004;8(3):273–92.

[9] Horowitz-Kraus T, Vannest JJ, Gozdas E, Holland SK. Greater Utilization of Neural-Circuits Related to Executive Functions is Associated with Better Reading: A Longitudinal fMRI Study Using the Verb Generation Task. Frontiers in human neuroscience. 2014;8:447 10.3389/fnhum.2014.00447 24999322 PMC4064667

[10] Giraud A, Poeppel D. Cortical oscillations and speech processing: emerging computational principles and operations. Nature neuroscience. 2012;15(4):511–7. 10.1038/nn.3063 22426255 PMC4461038

[11] Whitehurst GJ, Lonigan CJ. Child development and emergent literacy. Child development. 1998;69(3):848–72. . 9680688

[12] Kuhl P. Early Language Learning and Literacy: Neuroscience Implications for Education. Mind Brain and Education. 2011;5(3):128–42. 10.1111/j.1751-228X.2011.01121.x PMC3164118 21892359

[13] Horowitz-Kraus T, Hutton JS. From emergent literacy to reading: how learning to read changes a child's brain. Acta paediatrica (Oslo, Norway: 1992). 2015a;104(7):648–56. Epub 2015/04/08. 10.1111/apa.13018 . 25847632

[14] Sacchi E, Laszlo S. An event-related potential study of the relationship between N170 lateralization and phonological awareness in developing readers. Neuropsychologia. 2016; 91:415–25. 10.1016/j.neuropsychologia.2016.09.001 27614290

[15] Turkeltaub PE, Gareau L, Flowers DL, Zeffiro TA, Eden GF. Development of neural mechanisms for reading. Nature neuroscience. 2003;6(7):767–73. Epub 2003/05/20. 10.1038/nn1065 . 12754516

[16] Hutton JS, Horowitz-Kraus T, Mendelsohn AL, DeWitt T, Holland SK. Home Reading Environment and Brain Activation in Preschool Children Listening to Stories. Pediatrics. 2015;136(3):466–78. Epub 2015/08/12. 10.1542/peds.2015-0359 . 26260716 PMC9923605

[17] O'Farrellya C, Doylea O, Victorya G, Palamaro-Munsellc E. Shared reading in infancy and later development: Evidence from an early intervention. Journal of Applied Developmental Psychology. 2010;54:69–83.

[18] Bus AG, Van IJzendoorn MH, Pellegrini AD. Joint Book Reading Makes for Success in Learning to Read: A Meta-Analysis on Intergenerational Transmission of Literacy. Review of Educational Research. 1995;65(1):1–21. 10.3102/00346543065001001

[19] Crain-Thoreson C, Dal PS. Do Early Talkers Become Early Readers? Linguistic Precocity, Preschool Language, and Emergent Literacy. Developmental psychology. 1992;28(3):421–9.

[20] DeBaryshe BD. Joint picture-book reading correlates of early oral language skill. Journal of Child Language. 1993;20(2): 455–61 10.1017/s0305000900008370 8376479

[21] Sénéchal M, LeFevre JA, Hudson E, Lawson EP. Knowledge of storybooks as a predictor of young children's vocabulary. Journal of Educational Psychology. 1996;88(3):520–36.

[22] Derrfuss J, Brass M, Neumann J, Yves von Cramon D. Involvement of the Inferior Frontal Junction in Cognitive Control: Meta-Analyses of Switching and Stroop Studies. Human brain mapping. 2005;25:22–34. 10.1002/hbm.20127 15846824 PMC6871679

[23] Hutton J, Phelan K, Horowitz-Kraus T, Dudley J, Altaye M, DeWitt T, et al Shared Reading Quality and Brain Activation during Sto ry Listening in Preschool-Age Children. The Journal of pediatrics. 2017;191:204–12. 10.1016/j.jpeds.2017.08.037 29173308 PMC5728185

[24] Horowitz-Kraus T, Hutton J, Phelan K, Holland SK. Maternal reading fluency is positively associated with greater functional connectivity between the child's future reading network and regions related to executive functions and language processing in preschool-age children. Brain and cognition. 2018. 10.1016/j.bandc.2018.01.003 29316485

[25] Justice LM, Pullen PC. Promising interventions for promoting emergent literacy skills: Three evidence-based approaches. Topics in Early Childhood Special Education. 2003;23:99–113.

[26] Whitehurst GJ, Arnold DS, Epstein JN, Angell AL, Smith M, Fischel JE. A picture book reading intervention in day care and home for children from low-income families. Developmental Psychology,. 1994;30(5):679–89.

[27] Twait E, Farah R, Shamir N, Horowitz-Kraus T. Dialogic Reading Intervention in Preschoolers is Related to Greater Cognitive Control: an EEG Study. Acta Pediatrica. Accepted. 10.1111/apa.14841 31074876

[28] Kuhl PK RR, Bosseler A, Lin JF, Imada T. Infants' brain responses to speech suggest analysis by synthesis. Proc Natl Acad Sci U S A 2014;111(31):11238–45. 10.1073/pnas.1410963111 25024207 PMC4128155

[29] Kuhl PK, Tsao FM, Liu HM. Foreign-language experience in infancy: effects of short-term exposure and social interaction on phonetic learning. Proceedings of the National Academy of Sciences of the United States of America. 2003;100(15):9096–101. Epub 2003/07/16. 10.1073/pnas.1532872100 12861072 PMC166444

[30] Kuhl PK. Brain Mechanisms in Early Language Acquisition. Neuron. 2010;67(5):713–27. 10.1016/j.neuron.2010.08.038 20826304 PMC2947444

[31] Kuhl PK. Early language aquisition: cracking the speech code. NATURE REVIEWS, NEUROSCIENCE. 2010;831(5):831–43. 10.1038/nrn1533 15496861

[32] Dehaene-Lambertz G, Hertz-Pannier L, Dubois J, Meriaux S, Roche A, Sigman M, et al Functional organization of perisylvian activation during presentation of sentences in preverbal infants. Proceedings of the National Academy of Sciences of the United States of America. 2006;103(38):14240–5. Epub 2006/09/14. 10.1073/pnas.0606302103 16968771 PMC1599941

[33] Telkemeyer S, Rossi S, Koch SP, Nierhaus T, Steinbrink J, Poeppel D, et al Sensitivity of newborn auditory cortex to the temporal structure of sounds. The Journal of neuroscience: the official journal of the Society for Neuroscience. 2009;29(47):14726–33. 10.1523/JNEUROSCI.1246-09.2009 . 19940167 PMC6666009

[34] Holland SK, Vannest J, Mecoli M, Jacola LM, Tillema JM, Karunanayaka PR, et al Functional MRI of language lateralization during development in children. International journal of audiology. 2007;46(9):533–51. Epub 2007/09/11. 10.1080/14992020701448994 17828669 PMC2763431

[35] Sroka MC, Vannest J, Maloney TC, Horowitz-Kraus T, Byars AW, Holland SK. Relationship between receptive vocabulary and the neural substrates for story processing in preschoolers. Brain Imaging Behav. 2015;9(1):43–55. Epub 2014/12/24. 10.1007/s11682-014-9342-8 . 25533780

[36] Horowitz-Kraus T, Vannest JJ, Holland SK. Overlapping neural circuitry for narrative comprehension and proficient reading in children and adolescents. Neuropsychologia. 2013;51(13):2651–62. Epub 2013/09/14. 10.1016/j.neuropsychologia.2013.09.002 . 24029377

[37] Vannest JJ, Karunanayaka PR, Altaye M, Schmithorst VJ, Plante EM, Eaton KJ, et al Comparison of fMRI data from passive listening and active-response story processing tasks in children. Journal of magnetic resonance imaging: JMRI. 2009;29(4):971–6. Epub 2009/03/24. 10.1002/jmri.21694 19306445 PMC2763568

[38] Federmeier KD, Wlotko EW, Meyer AM. What's ‘Right’ in Language Comprehension: Event‐Related Potentials Reveal Right Hemisphere Language Capabilities. Lang Linguist Compass. 2008;1(2(1)):1–17. 10.1111/j.1749-818X.2007.00042.x PMC2748422 19777128

[39] Alday PM, Schlesewsky M, Bornkessel-Schlesewsky I. Electrophysiology Reveals the Neural Dynamics of Naturalistic Auditory Language Processing: Event-Related Potentials Reflect Continuous Model Updates. ENEURO. 2017;0311-16.2017. 10.1523/ENEURO.0311-16.2017 PMC5779117 29379867

[40] Luo H, Poeppel D. Phase patterns of neuronal responses reliably discriminate speech in human auditory cortex. Neuron. 2007;54(6):1001–10. 10.1016/j.neuron.2007.06.004 17582338 PMC2703451

[41] Plvermoller F, Birbaumer N, Lutzenberger W, Mohr B. High-Frequency Brain Activity: Its Possible Role in Attention, Perception and Language Processing. Progress in Neurobiology. 1997;52:427–45. 10.1016/s0301-0082(97)00023-3 9304700

[42] Klimesch W. EEG alpha and theta oscillations reflect cognitive and memory performance: a review and analysis. Brain Research Reviews 1999;29:169–19. 10.1016/s0165-0173(98)00056-3 10209231

[43] Anderson AJ, Perone S. Developmental change in the resting state electroencephalogram: insights into cognition and the brain. Brain and cognition. 2018;126:40–52. 10.1016/j.bandc.2018.08.001 30144749

[44] Perone S, Palanisamy J, Carlson SM. Developmental change in brain rhythms from early to middle childhood: Links to executive function. Developmental science. 2018;21(6):e12691 10.1111/desc.12691 29863816

[45] Başar E. A review of alpha activity in integrative brain function: fundamental physiology, sensory coding, cognition and pathology. International Journal of Psychophysiology. 2012;86(1)(6):1–24. 10.1016/j.ijpsycho.2012.07.002 22820267

[46] Cheung MC, Chan AS, Han YM, Sze SL. Brain activity during resting state in relation to academic performance. Journal of Psychophysiology. 2014;28(47-53).

[47] Başar E. A review of alpha activity in integrative brain function: fundamental physiology, sensory coding, cognition and pathology. International Journal of Psychophysiology. 2012;86(1):1–24. 10.1016/j.ijpsycho.2012.07.002 22820267

[48] Herrmann CS, Strüber D, Helfrich RF, Engel AK. EEG oscillations: from correlation to causality. International Journal of Psychophysiology. 2016;103:12–21. 10.1016/j.ijpsycho.2015.02.003 25659527

[49] Conners CK. Conners’ Kiddie Continuous Performance Test. North Tonawanda. NY: Multi-Health Systems; 2006.

[50] Wechsletr D. The Wechsler Preschool and Primary Scale of Intelligence, Third Edition (WPPSI-III). 2002.

[51] Shatil E. Shatil test for the assesment of early childhood literacy. Israel: Ach Publishers; 2000.

[52] Schmithorst VJ, Holland SK, Plante E. Development of effective connectivity for narrative comprehension in children. Neuroreport. 2007;18(14):1411–5. Epub Epub 2007/08/23. 10.1097/WNR.0b013e3282e9a4ef 00001756-200709170-00001. 17712265 PMC2762809

[53] Oostenveld R, Fries P, Maris E, Schoffelen JM. FieldTrip: Open Source Software for Advanced Analysis of MEG, EEG, and Invasive Electrophysiological Data. Computational Intelligence and Neuroscience. 2011;1:1–9. 10.1155/2011/156869 PMC3021840 21253357

[54] Oostenveld R, Stegeman DF, Praamstra P, van Oosterom A. Brain symmetry and topographic analysis of lateralized event-related potentials. Clinical Neurophysiology. 2003;114(7):1194–202 10.1016/s1388-2457(03)00059-2 12842715

[55] Mazziotta JC, Toga AW, Evans AC, Fox PT, Lancaster J, Zilles K, et al International Consortium for Brain Mapping. Four-dimensional probabilistic atlas of the human brain. Journal of the American Medical Informatics Association (JAMIA). 2001;8(5):401–30. 11522763 10.1136/jamia.2001.0080401 PMC131040

[56] Van Veen BD, van Drongelen W, Yuchtman M, Suzuki A. Localization of brain electrical activity via linearly constrained minimum variance spatial filtering. IEEE transactions on bio-medical engineering 1997;44(9):867–80. 10.1109/10.623056 9282479

[57] Barnes‐Davis ME, Merhar SL, Holland SK, Kadis DS. Extremely preterm children exhibit increased interhemispheric connectivity for language: findings from fMRI‐constrained MEG analysis. Developlmental Scoence. 2018 10.1111/desc.12669 PMC6193851 29659125

[58] Zalesky AFA, Bullmore ET. Network-based statistic: identifying differences in brain networks. Neuroimage. 2010;53(4):1197–207. 10.1016/j.neuroimage.2010.06.041 20600983

[59] Newman MEJ. The structure and function of complex networks. SIAM Rev. 2003;45:167–256.

[60] Latora V, Marchiori M. Efficient behaviour of small-world networks. Phys Rev Lett. 2001;87:198701 10.1103/PhysRevLett.87.198701 11690461

[61] Rubinov M, Sporns O. Complex network measures of brain connectivity: uses and interpretations. NeuroImage. 2010;52(3):1059–69. Epub 2009/10/13. 10.1016/j.neuroimage.2009.10.003 . 19819337

[62] Watts DJ, Strogatz SH. Collective dynamics of 'small-world' networks. Nature. 1998;393(6684):440-2 Epub 1998/06/12. 10.1038/30918 . 9623998

[63] Kuhl PK. Is speech learning 'gated' by the social brain? Developmental science. 2007;10(1):110–20. Epub 2006/12/22. doi: DESC572 [pii] 10.1111/j.1467-7687.2007.00572.x . 17181708

[64] Moon C, Lagercrantz H, Kuhl PK. Language experienced in utero affects vowel perception after birth: a two-country study. ACTA Paediatra. 2013;102(2):156–60. 10.1111/apa.12098 PMC3543479 23173548

[65] Kuhl PK, Conboy BT, Coffey-Corina S, Padden D, Rivera-Gaxiola M, Nelson T. Phonetic learning as a pathway to language: new data and native language magnet theory expanded (NLM-e). Phil Trans R Soc B 2008;363:979–1000. 10.1098/rstb.2007.2154 17846016 PMC2606791

[66] Kalashnikova M, Varghese P, Di Liberto GM, Lalor EC, Burnham D. Infant-directed speech facilitates seven-month-old infants’ cortical tracking of speech. Nature. 2018 www.nature.com/scientificreports. 10.1038/s41598-018-32150-6 PMC6137049 30214000

[67] Somsen RJM, Van-Klooster BJ, Van-der-Molen MW, Van-Leeuwen HM. Growth spurts in brain maturation during middle childhood as indexed by EEG power spectra. Biol Psychol 1997;44(187–209). 10.1016/s0301-0511(96)05218-0 9043653

[68] Reeves B, Thorson E, Rothschild ML, McDonald D, Hirsch J, Goldstein R. Attention to television: Intrastimulus effects of movement and scene changes on alpha variation over time. International Journal of Neuroscience. 1985;27: 241–55. 10.3109/00207458509149770 4044133

[69] Weinstein S, Appel V, Weinstein C. Brain activity responses to magazine and television advertising. Journal of Advertising Research. 1980; 20:57–63.

